# Response to: How the pandemic affected the frequency, type, and intensity of migraines in students

**DOI:** 10.51866/lte.787

**Published:** 2024-11-11

**Authors:** Selvakumar Kiruthika, Lee Fan Tan, Chai Nien Foo

**Affiliations:** 1 BPT, MPT (Neuro Sciences), Department of Physiotherapy, M. Kandiah Faculty of Medicine and Health Sciences, Universiti Tunku Abdul Rahman, Selangor, Malaysia. Email: kiruthika@utar.edu.my; 2 BE (Biomedical Engineering), PhD (Engineering), Department of Mechatronics and BioMedical Engineering, Lee Kong Chian Faculty of Engineering and Science, Universiti Tunku Abdul Rahman, Selangor, Malaysia.; 3 BSc (Food Science), MBA (General Management), PhD (Community Health) Department of Population Medicine, M. Kandiah Faculty of Medicine and Health Sciences, Universiti Tunku Abdul Rahman, Selangor, Malaysia.

**Keywords:** Migraine, COVID-19, Online questionnaires, Severity and intensity

**Dear editor,**

We appreciate the opportunity to further elaborate on the concerns raised regarding our recent publication entitled ‘Migraine symptoms and association of triggers, coping strategies and clinical characteristics with COVID-19 diagnosis among university students in Peninsular Malaysia: A cross-sectional study’.^1^ We value the feedback provided and are pleased to address the points mentioned.^2^

We appreciate the critical evaluation of our study design. While online questionnaires have certain inherent limitations, they offer a multitude of advantages. They facilitate an extensive reach and enhance accessibility, thus enabling the collection of data from a heterogeneous and geographically dispersed participant pool. Furthermore, this methodology is remarkably economical. The rapidity of data collection and subsequent analysis constitutes another significant advantage, as responses are processed immediately, thereby permitting the swift generation of results. Moreover, the online survey format affords considerable convenience for participants, who may respond at their own pace and within their favoured environments, potentially resulting in elevated response rates. The anonymity afforded by online questionnaires frequently promotes more candid responses, particularly concerning sensitive topics.

While it can be difficult to verify whether the designated respondent is the actual individual completing the survey, we implemented security protocols, such as unique login credentials, which aided us in affirming the identity of the participants. In terms of the accuracy and reliability of responses, online questionnaires contain a built-in validation system, which consists of requirements for specific categories of answers or the cross-verification of responses to uncover inconsistencies. While spontaneous questions in face-to-face interactions may not occur in online formats, we designed adaptive questions, where follow-ups were triggered based on prior answers, mimicking real-time conversation dynamics. In addition, we addressed concerns pertaining to the respondents’ cognitive and physical capabilities by creating user-friendly, accessible interfaces designed to accommodate various needs, including compatibility with screen readers or simplified layouts. Comprehensive instructions and trial runs (a pilot study among 20 participants) were also executed before the study to ensure that the participants fully understood the questions.

In our study, all participants experienced the onset of migraines before the pandemic. To clarify this, the questionnaire included a specific section where the participants were asked if they experienced migraines. If they responded affirmatively, they were then required to answer the following question: ‘When did you start experiencing these symptoms of migraine?’ This was followed by compulsory questions regarding COVID-19, asking the participants whether they had been diagnosed with COVID-19 since 2019 (with options of ‘yes’, ‘no’ or ‘not diagnosed with COVID-19 but have migraine symptoms’). These questions were designed to differentiate between pre-pandemic migraines and migraines potentially linked to SARS-CoV-2 infection, ensuring that the data reflected any differences in migraine manifestation related to COVID-19.

The ‘severity’ and ‘intensity’ of migraines were distinctly delineated within the questionnaire to capture separate dimensions of the migraine experience. ‘Severity’ pertained to the overall influence of migraine on daily activities and quality of life, which may encompass factors such as the duration of migraine or its debilitating effects. For instance, an individual may endure mild migraines that induce discomfort but do not significantly impede their daily routines, whereas at other times, they may confront more severe migraines that are profoundly disruptive, as illustrated by statements such as ‘I usually experience mild severity, but it has escalated over the past 1.5 years’. Conversely, ‘intensity’ was defined as the strength of the pain encountered during a migraine episode. This aspect pertained more to the physical pain experienced during an episode, which can range from mild to excruciating, and was quantified utilising a standardised pain scale.

The number of patients who were vaccinated versus those who were not was not reported because vaccination status was not assessed as part of the study. The primary emphasis of the questionnaire centred on the initiation, severity and intensity of migraine manifestations rather than on potential precipitating factors such as vaccination. It is recognised that SARS-CoV-2 vaccination may have the capacity to affect the onset of migraines or intensify pre-existing migraine conditions; however, this particular dimension fell outside the purview of our study. Future research could consider including vaccination status to explore its potential relationship with migraine characteristics.

Despite the absence of a control cohort of patients with migraine, the questionnaire was meticulously crafted to evaluate the frequency, intensity, duration and severity of migraines in relation to COVID-19 status. The participants were stratified according to their COVID-19 testing results: those who tested positive, those who had close contact with confirmed cases and those who had not been diagnosed with COVID-19 yet exhibited migraine symptoms. This methodological stratification facilitated a distinction among these three cohorts, yielding insights into the potential influence of the pandemic on migraine characteristics. Although the inclusion of a pre-pandemic control group would furnish a more direct comparative analysis, the design of this study serves to investigate the prospective effects of the pandemic and COVID-19 on migraine trends.

Migraine types was excluded from the parameters of the current investigation, which primarily concentrated on general migraine characteristics such as frequency, intensity and severity in relation to COVID-19 status, rather than differentiating among specific migraine subtypes. Furthermore, there were prospective plans established to evaluate the efficacy of physiotherapy interventions among patients with migraine, which may be influenced by COVID-19 status.

The paper addressed the number of participants who had suffered from COVID-19 in the results section, stating that 20 participants (11.5%) were diagnosed with COVID-19 via PCR testing as of October 2019, while 154 participants (88.5%) were not diagnosed. Among the 154 participants, 43 (27.9%) were suspected to have been in close contact with patients with COVID-19, while 111 (72.1%) had no diagnosis or known contact with patients with COVID-19 (non-diagnosed COVID-19 periods). The clinical characteristics (Table 2), migraine triggers (Tables 3 and 4) and coping strategies [coping strategies adopted by the participants diagnosed with COVID-19 included adequate sleep (65%), migraine medication (45%), acceptance (30%) and mental disengagement (15%)] were subsequently detailed.

Thank you for the time given to address these points and for the opportunity to further elaborate on the significance of the findings. We are grateful for the chance to contribute to this important discussion and hope this clarifies our perspective.
